# Environmental implications and evidence of natural products from dental calculi of a Neolithic–Chalcolithic community (central Italy)

**DOI:** 10.1038/s41598-021-89999-3

**Published:** 2021-05-21

**Authors:** Alessia D’Agostino, Gabriele Di Marco, Mauro Rubini, Silvia Marvelli, Elisabetta Rizzoli, Antonella Canini, Angelo Gismondi

**Affiliations:** 1 Department of Biology, University of Rome “Tor Vergata”, Via della Ricerca Scientifica 1, 00133 Rome, Italy; 2 Department of Archaeology, University of Foggia, Via A. Gramsci 89/91, 0881 Foggia, Italy; 3 Anthropological Service of SABAP-LAZ (Ministry of Culture Italy), Via Pompeo Magno 2, 00133 Rome, Italy; 4 Anthropological Laboratory of VA.VE. (Villa Adriana and Villa d’Este), Via degli Stabilimenti 5, Tivoli, 00019 Rome, Italy; 5 Laboratorio di Palinologia e Archeobotanica-C.A.A. Giorgio Nicoli, Via Marzocchi 17, 40017 San Giovanni in Persiceto, Bologna Italy; 6 PhD Program in Evolutionary Biology and Ecology, Department of Biology, University of Rome “Tor Vergata”, Rome, Italy

**Keywords:** Agroecology, Biodiversity, Community ecology, Evolutionary ecology, Anthropology, Cultural evolution, Plant domestication

## Abstract

In this contribution, we investigated the role of plants in the prehistoric community of *Casale del Dolce* (Anagni, FR, central Italy), through microparticles recovered from dental calculus. The finding of a great amount of pollen types, even in form of compact lumps, could indicate use of natural substances, such as honeybee products and/or conifer resins. This plant-microremain record also suggested environmental implications relative to the Neolithic and Chalcolithic period. Additionally, the stability of the tartar microenvironment had preserved starches and other microparticles, such as one epidermal trichome, a sporangium, and fragments of plant tissue, rarely detected in ancient dental calculus. The detection of secondary metabolites in the ancient matrix confirmed the familiarity of this community with plant resources. All these data supply various interesting food for thought and expand the knowledge about the potential of dental calculus in archaeological and archaeobotanical fields with a special focus on palaeoecology.

## Introduction

### Prehistorical and archaeological context

The spread of Neolithic culture in the Italian peninsula started from VI millennium B.C.^1^. The Neolithic transition began with populations which probably had come from the East. The first Neolithic cultural pattern arrived in the southern regions of Italy and moved northwards spreading along two sides: the Tyrrhenian and Adriatic coastlines. The impact of this phenomenon determined the onset of different bio-cultural innovations on both sides. While the Adriatic communities rapidly absorbed these new models, the Tyrrhenian ones (with reference to central Italy and specifically Latium) persisted for a long time in a condition still very similar to that of the last hunter-gatherers of the upper Palaeolithic. This is confirmed by the presence of so-called aceramic sites, populated by individuals morphologically very similar to the cro-magnonoid types, as in the case of Cisterna di Latina^2^. The advent of the Eneolithic culture in these territories produced a real cultural and biological revolution. In fact, the permeation of Gaudo's culture determined the introduction of a wider range of socio-economic innovations (e.g., changes in settlement structures, cultural assemblages, and mortuary practices), anatomical modernity, and further refinement of the encephalization process, the brachicrania^3,4^. In this regard, the site of *Casale del Dolce* (185 m.a.s.l., Anagni, Latium, central Italy) would represent the transition from the Neolithic to the Eneolithic period, with radiocarbon calibrated dates at 4.927 and 4.867 BP^5^, in which Gaudo’s culture is witnessed but brachycephalicalization not yet.

The archaeological context is characterized by inhabited and productive structures distributed on a series of terraces overlooking the Sacco River. The economic regime of this prehistoric community, settled in the valley from the Neolithic both for the fertile nature of the soil and for the relative proximity to the water source, would seem to be developed gradually out of a spectrum of well-established Neolithic subsistence practices.

Stable isotope signatures of several Neolithic and Eneolithic communities from central Italy would suggest a general sedentary lifestyle and a subsistence economy based on the procurement of local resources, with a general inclination to consume C_3_ plants and C_3_ consumer backbone resources. Interestingly, the same biomolecular approach has demonstrated that the individuals of *Casale del Dolce* could have a diet with a greater intake of carbohydrates compared to the others^6,7^. Additionally, more than 500 anthracological and carpological remains (e.g., *Carpinus* sp., *Quercus* sp., Pomoideae, *Corylus* sp.) were detected in the investigated site and analysed^5,8^. All this evidence would indicate that the Tyrrhenian community of *Casale del Dolce* was mainly devoted to agricultural practices and maximized the exploitation of the local environment, which had promoted its stabilization in the area. To corroborate this supposition, dental calculus analysis was performed on the human skeletal series of *Casale del Dolce*, addressing relevant aspects of the exploitation of plant and natural resources.

### Ancient plant diversity and dental calculus

Palaeobotany and archaeobotany are very informative topics of research which are characterized by interdisciplinary studies. In particular, the second one should be considered a valuable link between science and humanities. The scientific investigation on all types of plant remains, preserved both in natural and anthropogenic sediments, contributes to the comprehension of human–environment interaction and cultural diversity of our ancestors, in terms of evolution of phyto-associations, ecology, human impact, land exploitation, plant use, diet, and trades^9–13^.

So far, the study of plant and animal macroremains, the characterisation of organic residue in pottery, the analysis of stable isotopes, and the investigation of dental microwear have represented the most common methods to understand ancient habits. Nowadays, many research groups have exploited the great informative potential contained in ancient dental calculus to open a window on past lifeways. The recovery of microparticles trapped inside this matrix has been employing to infer new awareness about the impact of plants on different prehistoric and historical communities^14–23^. The interpretation of such type of results is still quite problematic and one of the major concerns occurs in establishing the authenticity of ancient particles, especially starch^24,25^. Due to the multifactorial aetiology of tartar and the individual variability of its growth, a linear correlation between plant consumption and presence of plant microremains in a calculus deposit does not exist. Additionally, how, when, and where different natural microdebris (i.e., from plants, minerals, and animals) have been trapped into the calculus remain quite challenging to assess^26^. As reported in literature^27^, several environmental particles, such as pollen, plant fibres, micro-charcoals, soot, soil particles, and dirt, grit and dust settled on food, can be also embedded in this matrix as a consequence of inhalation or accidental ingestion. Unambiguous interpretative scenarios are arduous to be defined for some types of microparticles, such as pollen and trichomes; thus, it is necessary to consider and critically explore all possible pathways of inclusion for them, beyond inhalation hypothesis. Published data mainly have focused on identifiable and generally diagnostic microparticles, such as starch granules. However, ancient calculus has revealed the capacity to preserve other microremains, such as trichomes, which would represent a direct link with the environment surrounding the individuals. For example, pollen can be considered as a bioindicator of plant diversity.

**Figure 1:**
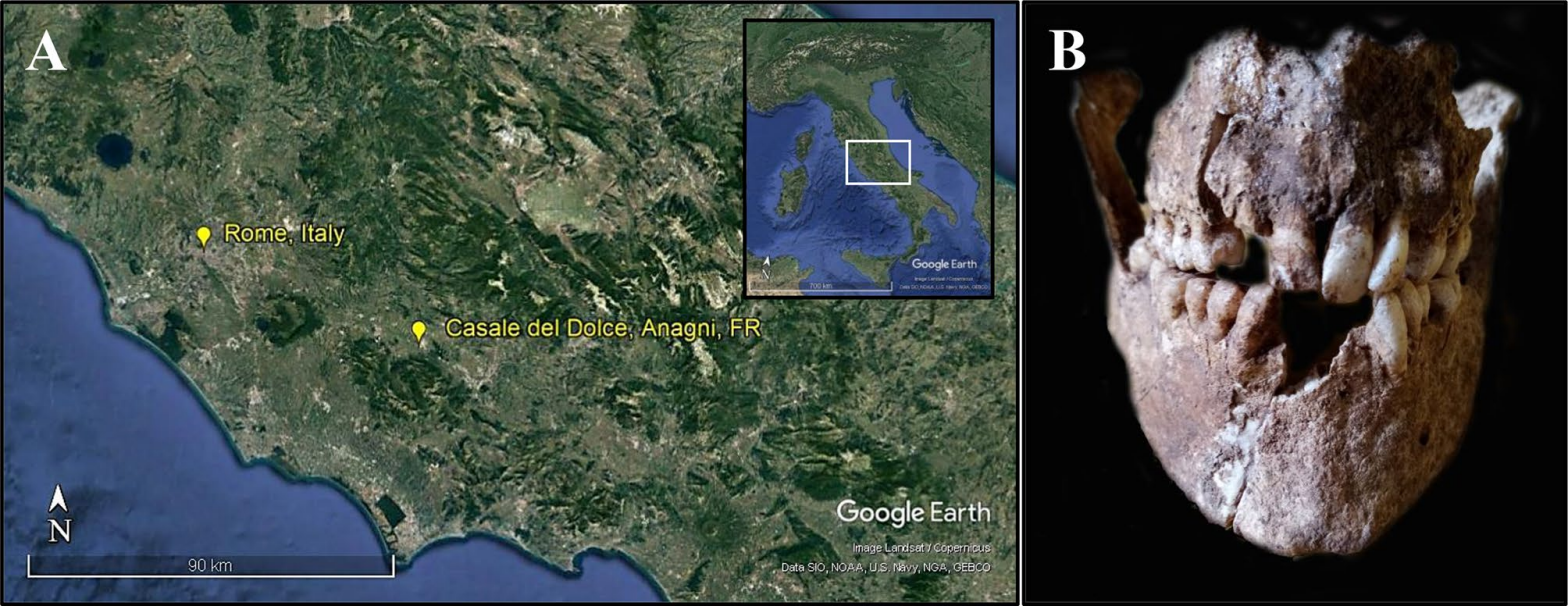
Archaeological site and masticatory apparatus of CDD7. Location of *Casale del Dolce* archaeological site (**A**): images produced using Google Earth Pro, V 7.3.3.7786 (June 25, 2019) *Casale del Dolce* (Latium, central Italy), 41° 41′ 47.43″ N, 13° 07′ 43.24″ E, Eye alt 184 m; Rome (Latium, Italy), 41° 54′ 10.02″ N, 12° 29′ 46.92″ E, Eye alt 47 m; Image Landsat/Copernicus, Data: SIO, NOAA, U.S. Navy, NGA, GEBCO, https://www.google.com/earth/ [Accessed September, 2020]; skeletal remains of CDD7 (image captured by Prof. Mauro Rubini) (**B**).

In this respect, our contribution aims at reconstructing the dietary ecology of the Neo-Eneolithic community of *Casale del Dolce* (Fig. 1). It also uses ancient dental calculus to deduce palaeoenvironmental implications, through the micro-botanical findings embedded in this archaeological repository.

## Results and discussion

### Morphological analysis

Food preparation or processing of plant material involve multiple activities and all of them can potentially leave micro-traces in the tartar, together with the environmental components.

Eleven dental calculi showed plant record: starches, pollen grains, one trichome, one sporangium, and tissue fragments (Table 1).

**Table 1:** Plant microdebris recovered from dental calculus samples of *Casale del Dolce*.

Lab code	Weight of calculus (g)	Starch Morphotype			Indeterminate starch	Total starches per sample	Single pollen grain	Pollen aggregates	Trichome	Fragment of plant tissues	Other microremains
		I	II	III							
CDD 1	0.019	2	1			3			1		
CDD 2	0.013	2				2	1P				
CDD 3	0.018		4			4					
CDD 4	0.021				1	1	1GP				
CDD 5	0.011					0					
CDD 6	0.021	1				1					
CDD 7	0.022	1				1	1A; 10B; 1C; 1Co; 2GC; 10GP; 1P; 7Q; 1T; 1U; 11ND	5		1	1SP
CDD 8	0.014	1				1					
CDD 9	0.041	1				1	1GC			2	
CDD 10	0.014	61				61					
CDD 11	0.013			1		1				3	
CDD 12	0.012	1	1			2					
Total		70	6	1	1	78	49	5	1	6	1

Amount and proposed identification of microparticles detected by light microscopy. Legend for pollen: A, *Alchemilla*-type; B, Brassicaceae; C, Cyperaceae; Co, *Corylus avellana*; Q, *Quercus* deciduous; GC, Cupressaceae; GP, Pinaceae; P, Poaceae spontaneous group; T, *Trifolium*-type; U, Urticaceae; ND, not determined; SP, sporangium. The weight of each dental calculus sample was reported in grams.

#### Plant hair

Trichomes are epidermal outgrowths characterized by different structure and function. Although plant hairs are some of the most common findings in the overall particulate matter carried by air (as pollen grains), in literature, only very few examples of trichomes in ancient contexts have been reported^28–30^. Trichome identification is not a common area in dental calculus research since they do not have a diagnostic morphology. For this reason, the identification of such type of microdebris must be based on realistic criteria, also in accordance with the geographical and historical context, providing all possible interpretative scenarios. The detection of trichomes in ancient tartar may disclose other lines of evidence than nutrition, representing a reliable archaeological environmental proof^31^.

**Figure 2:**
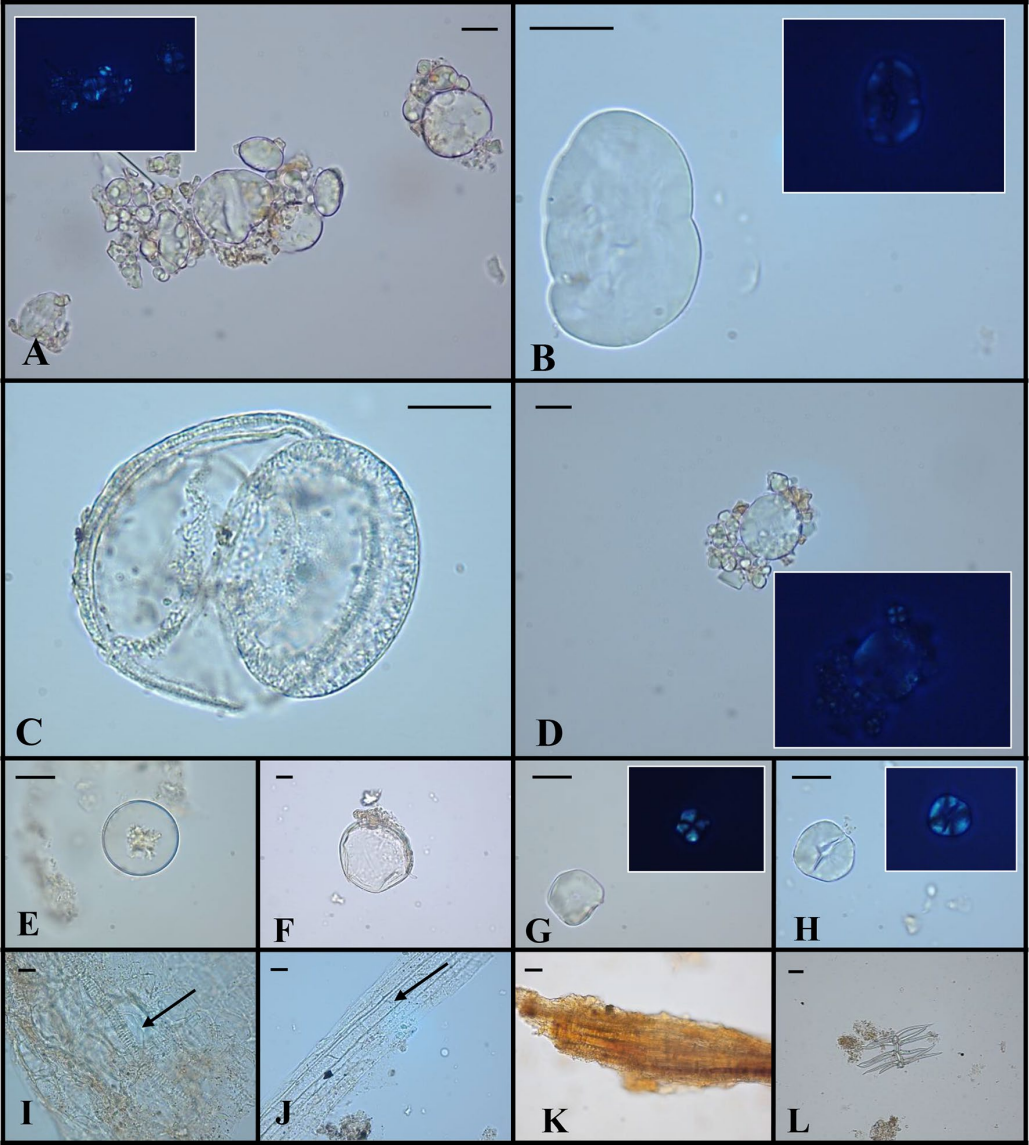
Plant microremains identified by light microscopy in dental calculus samples. Some of the images captured by optic microscopy were shown. Aggregate of Triticeae starch granules and relative polarized image (**A**); Fabaceae starch granule and relative polarized image (**B**); Pinaceae pollen grain (**C**); aggregate of Triticeae starch granules and relative polarized image (**D**); Cupressaceae pollen grain (**E**); Poaceae spontaneous group pollen (**F**); polyhedral starches of morphotype II (**G**,**H**); fragments of plant tissues (**I**–**K**); dendritic hair (**L**). The scale bar indicates 15 µm [45 µm for panel (**L**)]. Small flecks of calculus still attached to microremains can be observed in some panels.

One plant hair was identified in CDD1 sample (Table 1). This remain (Fig. 2L) falls into the general class of dendritic trichomes and its peculiar morphology has been more specifically termed *a candelabrum* or *abietiform*^32^. The overall structure corresponded to non-glandular and pluricellular trichomes with a central uniseriate axis and whorls of unicellular rays emerging at the joints of the axis. Usually, 4 *radii* from each node occurred perpendicular to the central axis. As exhaustively reported in literature, dendritic trichomes are known in ferns, different groups of modern monocots and basal eudicots, such as Scrophulariaceae and Platanaceae. Although dendritic, trichomes of ferns and monocots were excluded. Indeed, the first ones possess single secondary branches that alternatingly arise at an angle of 70°–120° with respect to the main axis along a single plane^33^, while the second ones show morphological features and appearance different from the ancient debris^34,35^. Candelabrum-like trichomes have been usually detected in *Verbascum* L. and *Platanus* L. species^36^. For this work, an experimental reference collection of trichomes from these plants was created (Supplementary Information 1). The general aspect of mullein trichomes appears to be capitate, bigger, more elongated, and slenderer than the microremain found in tartar sample. In addition, these trichomes seem to possess a pair of secondary elements per side or single secondary branches, which depart from the nodes, only rarely perpendicular to the central axis^37–39^. Thanks to the well-preserved morphology, the ancient candelabrum hair was interpreted as a *Platanus* sp. foliar trichome based on literature^40^ and our experimental reference, although mullein cannot be totally excluded. Dimensions, distance between nodes and the number of tapering secondary branches attached to the central axis of the microremain were like those of all plane species documented in literature^40–42^.

This finding leads to consider some paleoenvironmental implications. Fossil pollen analysis has demonstrated that, during the Plio-Pleistocene, Platanaceae were present in the Upper Valdarno (Italy)^43^. For the Holocene, likely as a consequence of Pleistocene glaciations, fragmentary and scarce evidence of plane tree have been found in Spain and French Mediterranean coast; no record of *Platanus* sp. has hitherto been found in Italy^44,45^. This thermophylous *taxon* has reappeared later as an ornamental tree, providing shade, during Roman times^46^. As we applied rigorous decontamination protocols, the evidence of this ancient trichome, probably accidentally inhaled by CDD1, may testify the presence of *Platanus* sp. and humid environments in central Italy during the Neo-Chalcolithic period.

#### Starch granules

More than 70 starches were retrieved from calculus samples (Table 1). Some of them were found in an extraordinary state of preservation, likely due to intentional ingestion and/or accidental inhalation during the processing of starchy foods. These grains were clustered in three different morphological types, based on the morphometric parameters (i.e., shape, size, presence of *lamellae* and *hilum*, aggregation level, and other secondary features) evidenced by literature. They were described using the International Code for Starch Nomenclature^47,48^.

*Morphotype I* These starches were consistent with those of Triticeae Dumort. tribe and occurred in almost all samples, as the most copious group (Table 1; Fig. 2A,D). Some grains were still lodged together. The morphotype was characterised by a bimodal distribution, or rather co-presence of large and small granules. Occasionally, the morphology was not completely intact, probably due to chewing as well as grinding and/or cooking procedures. These starch grains were similar to those occurring in caryopses of cereals, such as *Hordeum* sp. L. and *Triticum* sp. L. In particular, the diagnostic starches were oval to sub-round in 2D shape (15–43 µm in length; 10–35 µm in width). They had a central and distinct *hilum* and, sometimes, no visible *lamellae*. The small granules (≤ 10 μm in diameter) were spherical in shape with a central *hilum*. Knowledge about the Neo-Eneolithic period in central Italy is characterized by discontinuous data. The archaeobotanical dataset available for Latium is still limited^49^ but information about cultivated and wild-collected plants from *Casale del Dolce* site exists. In fact, the carpological analysis previously conducted^50^ has identified several caryopses of barley and wheat, supporting our results. The recovery of these starch grains, in almost all samples, suggested that the use of cereals was common and probably frequent for *Casale del Dolce* people, even if it is quite difficult to correlate presence of plant remains in calculus and quantity of consumed food^26^. The hypothesis of cereal consumption for this community has been also proposed by stable isotope data. Isotopic values would suggest a subsistence economy based on a great intake of carbohydrates and a lifestyle characterized by a progressive agricultural exploitation, even more evident than other Eneolithic sites of central Italy^6,51^. Lastly, Triticeae starches have been also found in dental calculus from Grotta dello Scoglietto (southern Tuscany), for the same pre-historical period^52^.

*Morphotype II* A low number of starch granules with faceted shape, perpendicular extinction cross and, sometimes, evident central fissures was recovered from dental calculus (Table 1; Fig. 2G,H). The morphology appeared oval to polygon (2D) with centric *hilum* and fissures radiating from it. The most frequent size distribution length was 14–25 μm in length and 13–17 μm in width. This type of grains exists in seeds of grasses belonging to the Andropogoneae Dumort. and Paniceae R. Br. tribes, as shown in the modern reference material^19^. Since an overlap in size and shape occurs among starches of species related to these tribes, the identification of these plant remains is arduous at a lower taxonomic level. *Sorghum* sp. Moench (sorghum), *Setaria* sp. P. Beauv. (foxtail millet) and *Panicum* sp. L. (millet) can be considered as potential candidates. Unfortunately, no phytolith, which would have helped us in distinguishing between the species of Paniceae^53^, was detected. In addition, the lack of an isotopic signal specific for this type of consumption and the absence of relative carpological remains for the archaeological site of *Casale del Dolce* might be due to a limited usage of these plants. In fact, although several species of these genera were diffused in Italy, little is known about their employment. The archaeobotanical evidence of millets (i.e., *Panicum* sp. and *Setaria* sp.) from the Late Neolithic period has been discussed; however, their cultivation is certain during the Bronze and Iron Ages^52,54,55^. Recently, Accelerator Mass Spectrometry-datings of prehistoric charred broomcorn millet grains has pinpointed the earliest occurrence of *Panicum miliaceum* L. in Europe at the middle of the 2nd millennium BCE (Middle/Late Bronze Age)^56^.

*Morphotype III* Only one grain contributed to the third type of starch (Table 1; Fig. 2B). It appeared to be consistent with the Fabaceae family, probably Vicieae (Bronn) DC. tribe (e.g., vetches) for its oval to elongated (irregular) shape and kidney-like. The *hilum* was obscured and sunken, while the *lamellae* were not fully visible. The size was 42 μm in length and 30 μm in width. Data about pulses are scarce for this period. In northern Italy, a high variety of pulses was already present in the Neolithic^57,58^ but this starch grain would seem to be one of the few and unique evidence of consumption in central and southern Italy. As this finding refers to a single individual, certainly, it is not expected to provide an exhaustive image of the use of pulses for the period and region but its presence, together with the carpological remains of Fabaceae^49,50^, could attest plant protein consumption.

A single starch granule was not classified because missing diagnostic and distinguishable characteristics. Probably modification events, such as grinding process, cooking procedure in water and/or chewing, and exposure to alfa-amylase, altered its shape.

#### Pollen grains

**Figure 3:**
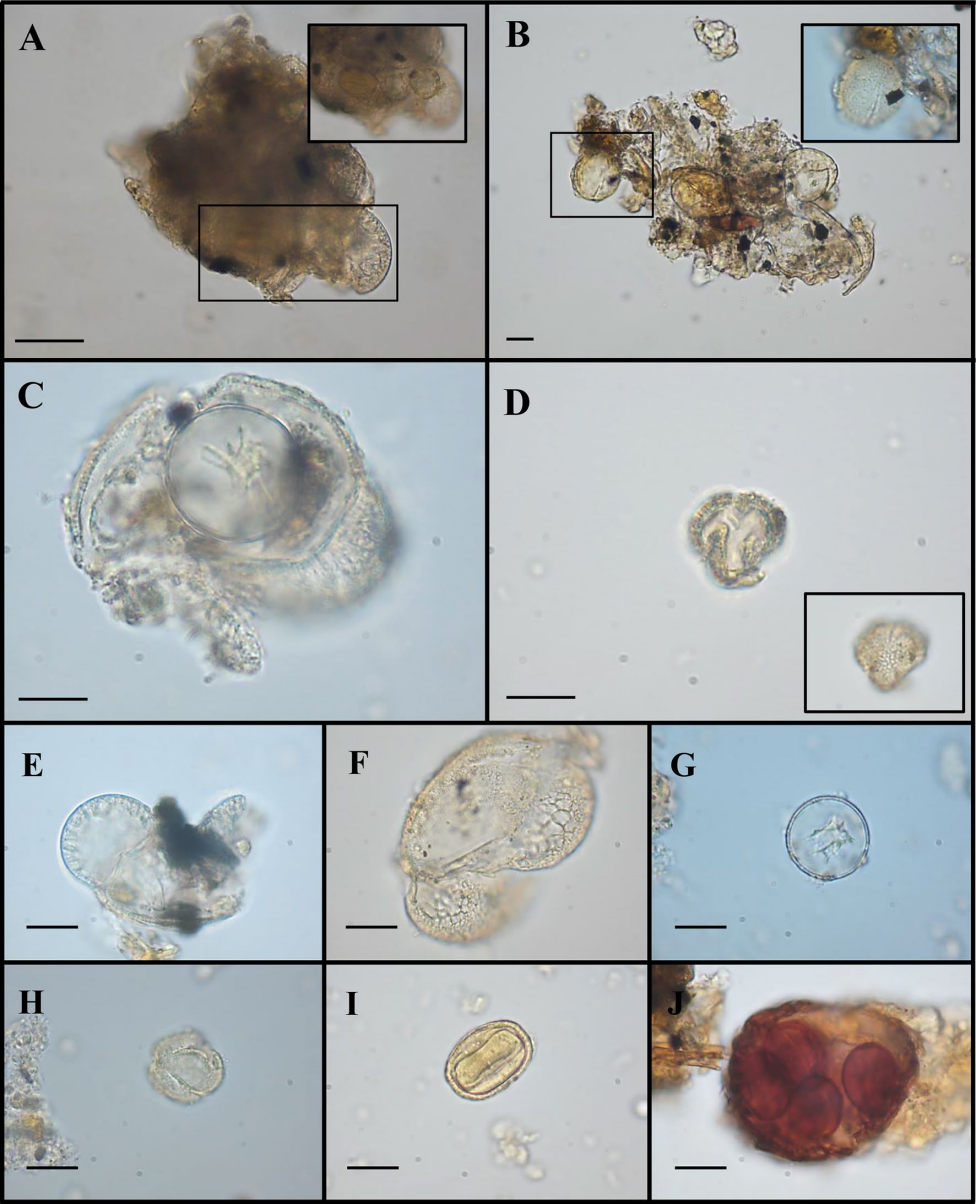
Plant micro-remains detected by morphological analysis in the dental calculus of CDD7 sample. Representative images obtained by light microscopy analysis were shown. Aggregates of pollen and spores (**A**,**B**); Pinaceae and Cupressaceae pollen grains (**C**); Brassicaceae pollen grain (**D**); Pinaceae pollen grains (**E**,**F**); Cupressaceae pollen (**G**); *Quercus* deciduous pollen (**H**); *Alchemilla* type pollen (**I**); sporangium of Monylophyta (**J**). The scale bar indicates 15 µm. Small flecks of calculus still attached to microparticles can be observed in some panels.

Four calculus samples showed the presence of different pollen types (Table 1). In total, 49 grains were found. Three of them were detected in CDD2, 4, and 9 (Fig. 2C,EF), while the remaining ones (46), both in single and in aggregate form, were retrieved from only one individual (CDD7) (e.g., in Fig. 3). All palynomorphs were identified according to morphometric parameters described in literature and evidenced in the Palynological Database^59^ and the names of the pollen types refer to literature^60–62^.

In this paragraph we describe the pollen grains found in CDD2, 4, and 9 samples.

The ancient microremain embedded in sample CDD2 was apolar and medium in size (63 µm in diameter), showing a morphology which typically occurs in Poaceae^63,64^. The stenopalynous nature of such type of pollen (that is, uniform monoporate) makes its systematic identification difficult. Although a low taxonomic determination limits paleoecological inferences, the evidence of Poaceae pollen is usually interpreted as indicative of open grasslands^65^.

One ancient palynomorph displayed morphological traits consistent with Pinaceae (sample CDD4). It appeared as a bisaccate monad with an elliptic corpus and medium reticulation on bladders^59,66,67^. Including sacci, the dimension was 56 µm in equatorial view.

A non-saccate Cupressaceae-type pollen, instead, was found in sample CDD9. It appeared spherical (with polar and equatorial axes of 30 µm) and inaperturate at light microscope; the protoplast exhibited itself star-like. Pollen grains produced by several species of Cupressaceae are considered morphologically uniform^68^. Since prehistoric times, Gymnosperm wood has been widely used as raw material and firewood, while needles, nuts and inner bark represented the edible parts of these trees^69^. Noteworthy is that the resins of these plants, possessing adhesive qualities and antibacterial properties, might have been also appreciated by Neanderthal^14^. Cupressaceae pollen grain is generally scarce in ancient sediments and one of the most underrepresented palynomorph in archaeological context. Several archaeobotanical studies have demonstrated the use of *Juniperus* L. species in the Mediterranean basin since the Holocene. In particular, the use of them as a source of aromatic foliage and resins employed for medicinal purposes, wood as fuel and for construction of dwellings, and fresh or dried berries as food has been proposed^70–72^. Sporadic fossil discoveries of *Cupressus* sp. L, instead, are rather sparse in the Mediterranean area, although some ancient record has been registered in Italy during the Quaternary^73^. Thus, the investigated plant microdebris testify the presence of Cupressaceae and provide additional evidence about the possible existence of evergreen Mediterranean forests, during the Neo-Chalcolithic period, in the Sacco River Valley.

#### Pollen grains in CDD7

CDD7 specimen (Fig. 1B), an adult male affected by severe malocclusion, preserved an interesting set of microparticles at microscopic analysis; therefore, we decided to report and discuss separately the data obtained from his calculus.

Eleven pollen grains out of 46 were not distinguishable due to the lack of diagnostic characteristics. The remaining 35 were found (singly, in pairs, or aggregates; Table 1, Fig. 3) in good or excellent state of conservation. The latter appeared as clusters of Pinaceae pollen (Gymnosperm) and other palynomorphs, including spores. Examples are shown in panels A and B of Fig. 3.

Two Cupressaceae, ten Pinaceae and one Poaceae pollen, presenting the same morphological features described in the previous paragraph, were also found in this sample (e.g., see Fig. 3C,E,F,G).

In addition, pollen grains from four herbaceous plants, namely Cyperaceae, Urticaceae, *Trifolium,* and *Alchemilla* species, and from the arboreal genus *Corylus* L. were detected and aredescribed below. Although pollen morphological variation within Cyperoideae subfamily is notable, one ancient microremain, possessing a pear-shape and a scabrate sculpture on its surface, appeared belonging to the genus *Carex*^74,75^. In equatorial view it was triangular and the polar axis length was 41 µm. A second pollen grain was recognised as Urticaceae-type; it exhibited spheroidal shape (equatorial diameter 23 μm) and scabrate ornamentation. This morphology occurs both in *Parietaria* sp. and *Urtica* sp. pollen grains^59,62^ and it is very difficult to distinguish them by optical microscope, especially if degraded. The shape of a third ancient monad, attributed to *Trifolium*-type (Fabaceae), was subprolate in equatorial view (46 μm) with scabrate ornamentation^76^. The *Alchemilla*-type (Rosaceae) microremain (26 μm equatorial view, Fig. 3I) was radially symmetrical, elliptic and prolate in shape^77^. Finally, another pollen type was found and attributable to *Corylus* sp. L. (Betulaceae). It was oval in equatorial view (19 μm), smooth, and tripolar with deep *oncusis* in each pore^78^.

Seven pollen grains were single, prolate, isopolar, and elliptic in equatorial view (polar axis 19–25 µm long). They were tricolpate, with long and narrow *colpi*. Pores were at times indistinct. Pollen of the different species of Fagaceae shows a high variability in form, size, sculpturing; for this reason, most of them overlap in morphology. The ancient palynomorphs in exam were closely similar to a *Quercus*-type (examples in Fig. 3A,H)^79,80^.

The last 10 grains showed a morphology (3-colpate, reticulate and subprolate) ascribable to Brassicaceae pollen grains (example in Fig. 3D). This is a stenopalynous family in which pollen varies among the genera but rarely in the species under the same genus^81,82^.

Intriguingly, pollen findings in sample CDD7 were numerous and deriving also from insect-pollinated plants (e.g., Brassicaceae). This evidence appeared like a honey palynospectrum. This type of assemblage has been never registered in dental calculus deposits and, especially for the aggregates, the hypothesis of accidental inhalation seems implausible. Precisely, the presence of aggregates induced us to reflect upon a common origin of the whole pollen record. However, for single granules, to date, the supposition of aspiration cannot be completely excluded, due to the multiple pathways of inclusion of such type of microparticles^27^. The high pollen variety could be explained by the presence of residues of natural matrices, as well as honey or beehive products (e.g., wax, propolis), in the calculus sample. To support our hypothesis, we prepared a reference collection based on modern multifloral honey samples (Supplementary Information 1, panel E–J).

Archaeological finds of bee products are quite rare^83–88^. Since the end of the upper Palaeolithic, honey has been employed as sweetener, while beeswax for technological, ritual, cosmetic and medicinal applications^89,90^. Regarding the latter, Bernardini et al.^91^ have found fascinating traces of a filling with beeswax, highlighting Neolithic dentistry procedures. It is important to recall that bees may also visit non-nectariferous plants (e.g., Poaceae, Betulaceae like *Corylus* sp.) for collecting pollen as protein source. Moreover, Pinaceae (*Pinus* sp. L. and *Abies* sp. Mill.) and Fagaceae (*Fagus* sp. L. and *Quercus* sp. L.), among others, emit sweet secretions and may be classified as honeydew producers^88^. Therefore, it is not unlikely to discover pollen grains of pine, hazel, oak, and cereals mixed with melliferous *taxa*. In fact, similarly, Carboni et al.^92^ have observed a lump of pollen inside an Eneolithic vessel, suggesting the use of a fermented honey-based drink, the mead, for ritual purposes.

According to all this evidence, the pollen record detected in the present ancient calculus could be likely interpreted as direct honey consumption and/or remain of food or beverage including honey as natural sweetener. However, the use of conifer resins as antimicrobial or flavouring agents, mixed to honey or alone, cannot be excluded, together with the hypothesis of inhalation of bisaccate pollen from the immediate environment.

Unfortunately, for the investigated site, no evidence supporting the previous hypotheses exists. Nevertheless, it is possible that the individuals from *Casale del Dolce* practised bee-keeping culture near woodland pastures, although this interpretation cannot be definitive.

Currently, pollen spectra from beehive products are used to deduce plant biodiversity of the areas visited by insects for nectar collection^93,94^. Bearing in mind this indication and the typical habitats of the identified plant *taxa*, some ecological implications were inferred. A thermophilic broad-leaved forest mainly made up of conifers (such as *Pinus*) and several deciduous trees (such as *Quercus* and *Corylus*), together with wet grasslands (Cyperaceae, Urticaceae, *Alchemilla* sp.), was outlined by pollen analysis. This hypothesis would seem consistent with Coubray’s work^50^, who has identified the wood charcoals found in the archaeological site of *Casale del Dolce* as *Carpinus* L., *Quercus*, Maloideae, *Cornus* L., *Corylus*, *Ulmus* L., *Fraxinus* L., and *Acer* L. remains. In addition, palynological analyses performed in the same region^95–97^ have detected similar vegetational elements.

#### Other plant microremains

We detected an unusual range of microparticles, that is, fragments of plant tissues and a sporangium, rarely documented in human dental calculus investigations (Table 1)^69,98–100^.

Among the first, one microparticle was made up of plant cells associated to a scalariform xylem vessel (Fig. 2I), while another debris showed wood cells with simple pits (Fig. 2J). A brown-yellowish fragment was also photographed (Fig. 2K). As reported in literature^99^, no evidence of charring or burning may be attributed to this type of darkening colouring but, if so, it would suggest an involuntary inhalation of ash particles from trees or shrubs used for fire. Thus, this type of microremain could derive from both non-edible and edible plants. In general, all these fragments retrieved from calculus might be the result of some activities, such as chewing of fresh plant organs, food and/or other uses of bark, oral hygiene procedures with woody dental picks, and/or use of teeth as a third hand^99,101,102^.

The second type of uncommon microparticle, found in sample CDD7 (Table 1), appeared morphologically like a sporangium, probably from Monylophyta (Fig. 3J). It was brownish in colour and ovoid in shape. This type of microremain has never been observed in so ancient human dental calculus. A more specific taxonomical identification is very complex and would be risky, since at palaeobotanical and/or archaeological level there is no evidence to support this finding. However, considering that sporangia are typically attached to the abaxial surface of the leaf and that airborne dispersal capability of fern spores into stronger wind currents is rare and improbable^100,103^, the recovery of the whole sporangium allowed us to hypothesize a voluntary use of fern leaves.

### Biochemical analysis

GC–MS approach revealed the presence of organic compounds derived from the matter ingested and/or inhaled by the individuals. However, the potential of the biomolecular approach on dental calculus is still highly challenging and the capacity to trace the origin of some molecules is still difficult, due to the multifactorial dental calculus’s aetiology^31,104^.

In Supplementary Information 2 (SI2), the molecules detected in each sample were listed and clustered in chemical classes. The chromatographic profiles were dominated by a series of C_6_ to C_30_ *n*-alkenes and *n*-alkanes, not reported in SI2 because ubiquitous and not taxonomically specific. They could probably come from degradation of oral bacteria or consumed food, representing, for instance, fragments of unsaturated or saturated lipids^14,105–107^.

The typology of residues accumulated in dental calculus and their adsorption capacity determine the lipid profile of this matrix, considering that different foods naturally possess variable lipid composition. For this reason, it is difficult to associate fatty acids to specific dietary sources. The presence of fatty acids (e.g., odd, short, and long chains), ubiquitous components of organic matter, could be considered indicator for consumption of animal fats or plant oils (e.g., oil-rich seeds and fruits)^104,108–113^. Long-chained polyunsaturated fatty acid derivatives (PUFAs; e.g., eicosapentaenoic acid, EPA), abundant in dried fruits^114^, were detected in some samples. Polyunsaturated omega-3 fatty acids have been rarely identified in archaeological contexts^115^, due to their highly inclination to oxidative alteration^116^. However, dental calculus has shown itself conservative for this type of molecules^31^. The consumption of aquatic organisms cannot be excluded, being rich of PUFAs^114^ and considering the proximity of the ancient settlement to the Sacco River.

Monoterpene derivatives, non-specific compounds with volatile nature, retrieved from some samples, such as citronellol, menthol and pinanol (commonly found in leaves, fruit, and bark of a wide range of plant species), could generically indicate the ingestion of plant materials or waxes^109^.

In CDD5 calculus, azulene and coumarin derivatives were also recovered. These secondary metabolites usually occur in species belonging to Apiaceae, Asteraceae, and Rutaceae families, well known medicinal plants possessing a wide range of biological activities^117,118^. As suggested by Hardy et al.^14^, the plant species rich in such type of bitter-tasting compounds might have been ingested for self-medication.

Two alkaloids were found: trigonelline and hordenine, respectively, in CDD4 and CDD7 specimens. The first one, whose accumulation takes place in various plant species (i.e., *Achillea* sp. L.) and especially in Fabaceae seeds (e.g., *Trigonella* sp. L., *Trifolium* sp. L., and *Medicago* sp. L.)^119,120^, might represents a further proof for consumption of pulses.

Hordenine, which naturally occurs in certain grasses, like cereals (e.g., barley, millet, and sorghum)^121^, could demonstrate the ingestion of starchy material, as already testified by the detection of a Triticeae starch granule in the same calculus flakes and the recovery of caryopses at the site^50^.

## Conclusions

To date, the complex correlation between ancient dental calculus and environmental flora biodiversity has not been fully understood and exploited yet. Thus, the main goal of the current study was the expansion of this issue. The high pollen variety observed in our pre-historic calculus samples pushes forward this field of research, allowing to hypothesize that the identified species were of local origin. These data would permit us to deduce some floristic-vegetational implications during the Neolithic and Chalcolithic period in central Italy. The simultaneous presence of traces of Poaceae and Fabaceae outlines a very important combination from a nutritional point of view, since cereals, rich in carbohydrates, are complementary to legumes, rich in proteins, and together creating a balanced and complete diet. Moreover, combining morphological approach with analytical chemistry, we provided an extensive body of evidence about the strong human–environment relationship of *Casale del Dolce* site. Although restricted to a limited series of samples, dental calculus analysis allowed us to confirm that these prehistoric individuals were comfortable with plants and able to exploit natural matrixes, offering the opportunity to maximize the biographical detail of this ancient population. To date, dental calculus represents one of the best archaeo-anthropological tools to highlight the value of plants in human evolution and culture, although it is still difficult to provide a univocal interpretation for some microremains embedded in it. The application of Next Generation Sequencing analysis on the ancient plant DNA extracted from this matrix could open up new and interesting interpretative scenarios.

## Methods

### Calculus sampling and analytical procedures

Twelve Neo-Eneolithic human skeletal remains (radiocarbon calibrated dated at 4.927 year. BP., Beta Analytic), hosted and preserved at the storerooms of the Villa Adriana and Villa d' Este (VA.VE.) (located in the Archaeological area of Santuario di Ercole Vincitore, Tivoli, Rome), were examined for dental calculus. Light deposits were detected above the gingival margin and removed from tooth enamel by an autoclaved dental pick. The mineralized flakes were collected separately on an aluminium foil and placed in sterilized micro-centrifuge tubes. The samples were then transferred to the Department of Biology of the University of Rome ‘Tor Vergata’ (Italy) for analysis.

Meticulous sterilisation and decontamination procedures were performed following our lab standard methods^31^. Here, laboratory contamination checks were regularly carried out on all workspaces, and supplies. Fresh disposable consumables (e.g., centrifuge tubes) and instruments (e.g., glassware, microscope slides, cover slip, metal tools) were autoclaved for 2 h, immediately prior to use. The absence of plant micro-residues in laboratory reagents and materials was also monitored. This intensive cleaning regime was applied with the same accuracy already reported in literature^20,31,122,123^.

Before proceeding with the preparation of dental calculus sample, the soil still adhering to the external part of the mineralized plaque was gently removed using a stereomicroscope (Leica ZOOM 2000, Leica, Buffalo, NY, USA) and a fine sterile acupuncture needle, under a sterile vertical laminar flow hood^31^. Afterward, calculus was treated by UV light for 10 min, immersed in 2% sodium hydroxide for 15 min, washed twice with sterilised water and dried at 37 °C. Preceding the cleaning procedure, six randomly selected dental calculi were washed by sterile water, which was examined by optical microscopy, to confirm the efficacy of the method. No microdebris was detected by light microscopy in the samples after decontamination.

To obtain the highest amount of information from these samples, the protocols used in our lab^20,31,122,123^ were applied, after some minor modification. For each individual, the mineralized plaque retrieved from different teeth was combined into a single tube where, after the decontamination procedures, 0.5 mL of 0.2 M hydrochloric acid was added and left to act for 24 h. Once solubilized, the sample was incubated with 0.5 mL of hexane, in agitation, for two hours. After centrifugation at 11,000*g* for 5 min, the supernatant fraction was recovered, dried out and, later, subjected to gas-chromatographic mass-spectrometry (henceforth GC–MS) analysis (see details below). The pellet resulting from the decalcification step was used to extract microparticles and perform the morphological analysis by optical microscopy (henceforth OM). In detail, this pellet was washed three times with ultrapure water and mounted on a glass slide in a water-glycerol solution (1:1, v/v). The sample was observed through an optical microscope (ZEISS Axio Observer 7, Zeiss, Jena, Germany) equipped with polarized filters and Zen imaging software 2.6, operating at different magnifications. The recovered microdebris were identified on morphometric features, in comparison to literature and our modern reference materials^19,48,124^. The starches were described using the International Code for Starch Nomenclature^47^.

GC–MS analysis was performed using a QP2010 system (Shimadzu, Kyoto, Japan), in triplicate on each dental calculus sample. The pellet obtained from the hexane fraction was resuspended in 60 µL of hexane and derivatized with 40 µL of Methyl-8-Reagent (Thermo Scientific, Bellefonte, PA, USA). Two microliters of extract were injected into the instrument at the temperature of 280 °C, in splitless modality. The sample was separated by an SH-Rtx-5MS capillary column (Shimadzu; length 30 m × diameter 0.25 mm × thickness 0.25 μm). The carrier gas was helium, employed at a constant flow of 1 mL/min. The temperature gradient was set as follows: 60 °C for 5 min, 150 °C for 5 min (reached at a rate of 6 °C/min), 250 °C for 5 min (reached at a rate of 6 °C/min), and 300 °C for 5 min (reached at a rate of 6 °C/min); to obtain a better resolution. An electron impact of 70 eV (scanning from 100 to 700 *m/z*) was used for the ionization (ion source temperature 230 °C; interface temperature 320 °C; solvent cut time 6 min). The detected molecules were identified by comparing their mass spectra with those registered in the software database NIST (National Institute of Standards and Technology) Library 14 and on-line support^125^. Scientific food databases and literature data were consulted for reconstructing food categories and plant species.

### Experimental reference

Some of the microdebris (i.e., trichomes, pollen grains) found in the dental calculus were compared to a reference collection composed of plant microremains extracted from modern species and honeys. The reference collection was prepared in a separated laboratory. Trichomes of *Verbascum sinuatum* L. and *Platanus* sp. L. were collected with a drop of ultrapure water, mounted as previously described for calculus samples, and observed at light microscope. Pollen residue from honey was obtained by resuspending the bee product (5 g) in ultrapure sterilized water (15 mL) and centrifuging it at 7830*g* for 10 min. This procedure was repeated twice, and the final pellet was dissolved in fuchsine and analysed by OM.

## Supplementary Information

Supplementary Information 1.

Supplementary Information 2.

## Abbreviations

GC–MS: Gas chromatography mass spectrometry
OM: Optic microscopy
