# Supplementary material for: Environmental implications and evidence of natural products from dental calculi of a Neolithic–Chalcolithic community (central Italy)
Source: Sci Rep. 2021 May 21;11:10665. doi: 10.1038/s41598-021-89999-3 (PMC8140145; doi:10.1038/s41598-021-89999-3)

Environmental implications and evidence of natural products from dental calculi of a Neolithic-Chalcolithic community (central Italy) - Alessia D'Agostino, Gabriele Di Marco, Mauro Rubini, Silvia Marvelli, Elisabetta Rizzoli, Antonella Canini and Angelo Gismondi

SUPPORTING INFORMATION 1. Experimental collection. Representative images from modern materials used as reference: trichomes of *Platanus* sp. (A-B); trichomes of *Verbascum sinuatum* L. (C-D); honey pollen residues (E-J). The scale bar indicates 30 µm.

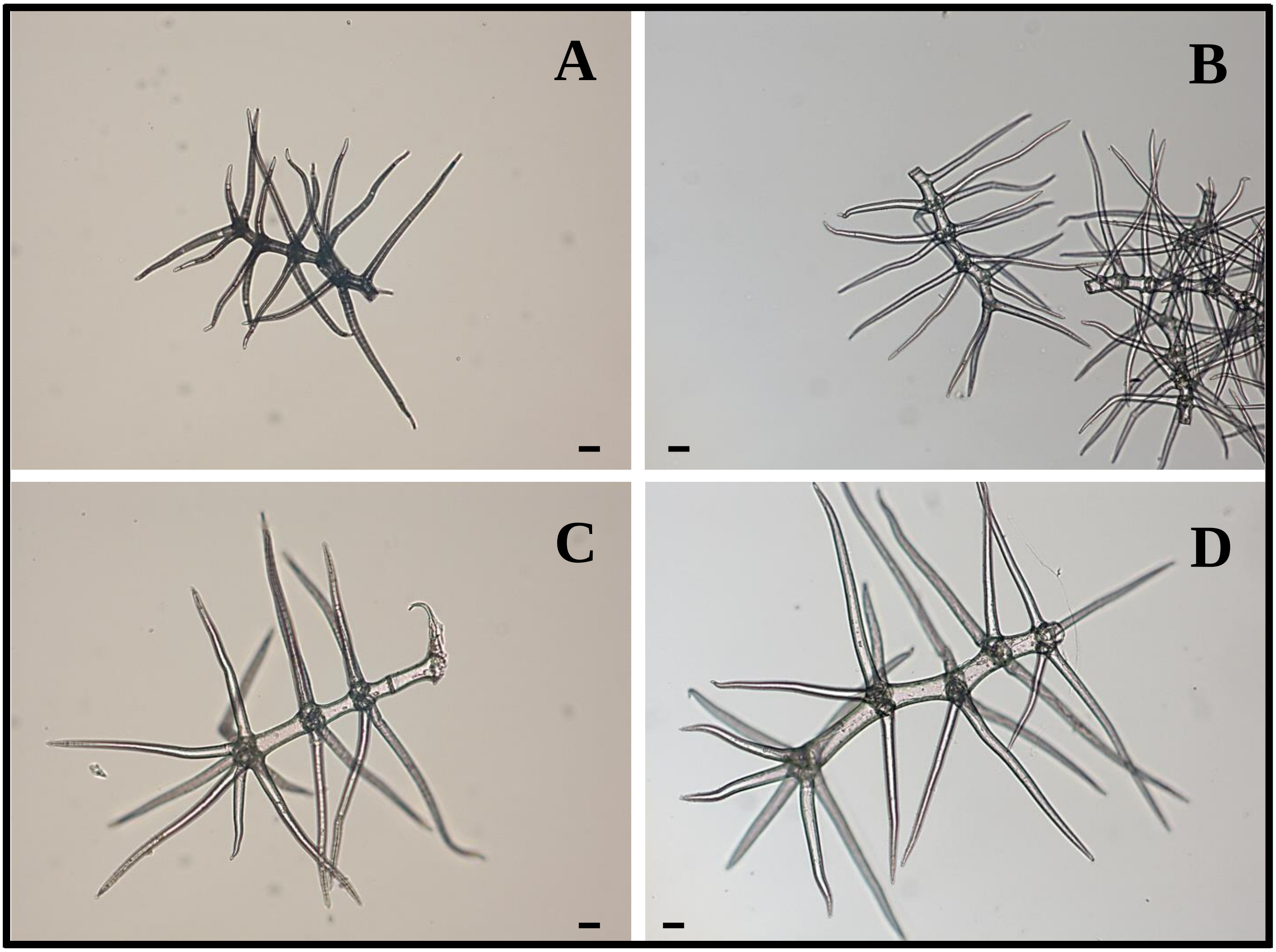

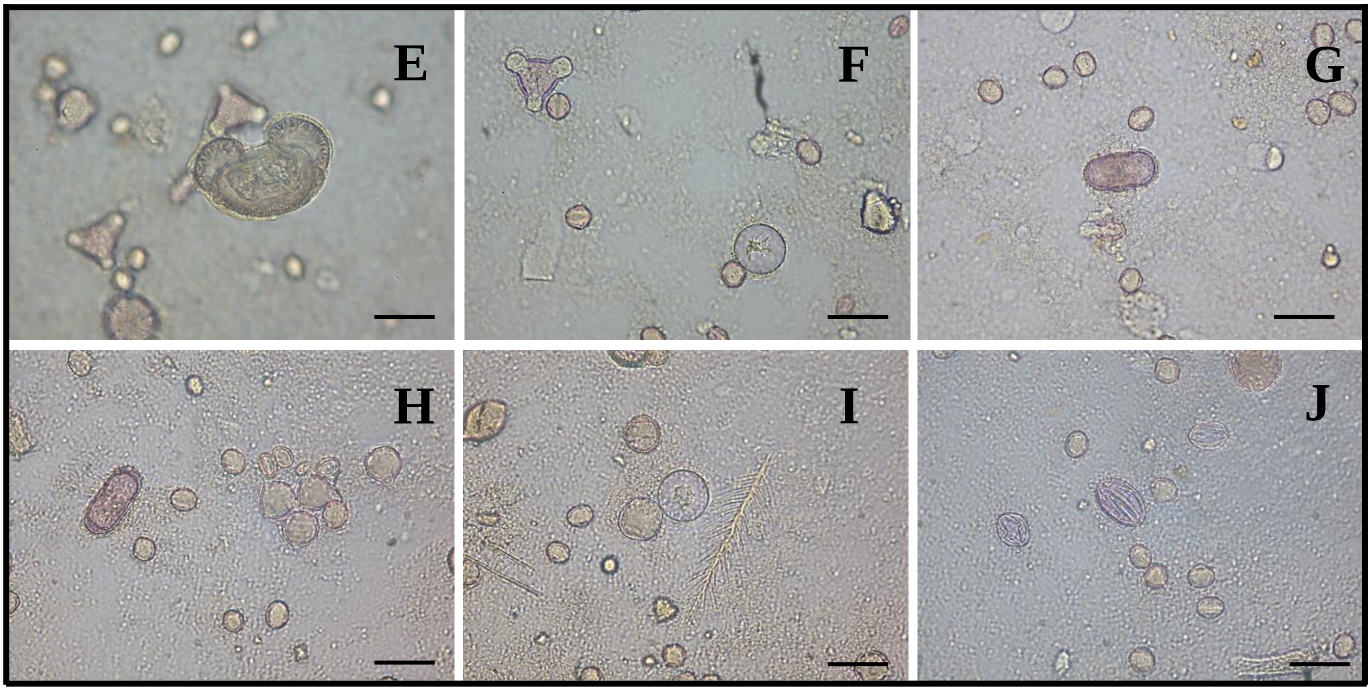

Supplement: Supplementary file 1 — Supplementary Information 1. [file 41598_2021_89999_MOESM1_ESM.pdf]
