# Supplementary material for: Environmental implications and evidence of natural products from dental calculi of a Neolithic–Chalcolithic community (central Italy)
Source: Sci Rep. 2021 May 21;11:10665. doi: 10.1038/s41598-021-89999-3 (PMC8140145; doi:10.1038/s41598-021-89999-3)
Supplement: Supplementary file 2 — Supplementary Information 2. [file 41598_2021_89999_MOESM2_ESM.pdf]

Environmental implications and evidence of natural products from dental calculi of a Neolithic-Chalcolithic community (central Italy)

Alessia D'Agostino, Gabriele Di Marco, Mauro Rubini, Silvia Marvelli, Elisabetta Rizzoli, Antonella Canini and Angelo Gismondi

SUPPORTING INFORMATION 2. Chemical markers identified in dental calculus. List of chemical compounds detected by GC-MS, excluding n-alkenes and n-alkanes.

CDD1			
Fatty acids	Saturated		Pentanoic acid; Dodecanoic acid; Tetradecanoic acid; Hexadecanoic acid; Octadecanoic acid; Heneicosanoic acid; Tetracosanoic acid; Pentacosanoic acid
	Unsaturated	omega-6	9,12-Octadecadienoic acid
		omega-7	11-Octadecenoic acid
		omega-9	9-Octadecenoic acid
Alcohols			1-Dodecanol; 9-Octadecen-1-ol
Terpens and terpenoids	Monoterpenes and derivatives		Citronellol

CDD 2			
Fatty acids	Saturated		Tetradecanoic acid; Hexadecanoic acid; Octadecanoic acid; Nonadecanoic acid; Heptacosanoic acid; Triacontanoic acid
	Unsaturated	omega-6	9,12-Octadecadienoic acid
		omega-9	9-Octadecenoic acid

CDD 3			
Fatty acids	Saturated		Dodecanoic acid; Tetradecanoic acid; Hexadecanoic acid; Octadecanoic acid; Nonadecanoic acid; Pentacosanoic acid
	Unsaturated	omega-3	2,6,10,14,18-Pentamethyl-2,6,10,14,18-eicosapentaene
		omega-6	9,12-Octadecadienoic acid
		omega-7	9-Hexadecenoic acid; 11-Octadecenoic acid
		omega-9	9-Octadecenoic acid
Terpens and terpenoids	Monoterpenes and derivatives		Citronellol

CDD 4			
Fatty acids	Saturated		Dodecanoic acid; Tridecanoic acid; Hexadecanoic acid; Octadecanoic acid
	Unsaturated	omega-3	2,6,10,14,18-Pentamethyl-2,6,10,14,18-eicosapentaene
		omega-6	9,12-Octadecadienoic acid
Alcohols			9-Octadecen-1-ol
Alkaloids and derivatives			Trigonelline

Terpens and terpenoids	Monoterpenes and derivatives	Citronellol; Menthol

CDD 5			
Fatty acids	Saturated		Tridecanoic acid; Hexadecanoic acid; Octadecanoic acid
	Unsaturated	omega-6	9,12-Octadecadienoic acid
		omega-7	11-Octadecenoic acid
Alcohols			9-Octadecen-1-ol
Terpens and terpenoids	Sesquiterpenes and derivatives		1,4-Methanoazulen-7(1H)-one, octahydro-4,8,8,9-tetramethyl-
Phenolic compounds and derivatives			Coumarin, 3,4-dihydro-4,5,7-trimethyl-

CDD 6			
Fatty acids	Saturated		Tetradecanoic acid; Hexadecanoic acid; Octadecanoic acid
	Unsaturated	omega-3	2,6,10,14,18-Pentamethyl-2,6,10,14,18-eicosapentaene
		omega-6	9,12-Octadecadienoic acid
		omega-7	11-Octadecenoic acid
		omega-9	9-Octadecenoic acid; 15-Tetracosenoic acid
Alcohols			1-Dodecanol; 9-Octadecen-1-ol

CDD 7			
Fatty acids	Saturated		Hexadecanoic acid; Octadecanoic acid
	Unsaturated	omega-6	9,12-Octadecadienoic acid
		omega-9	9-Octadecenoic acid
Alkaloids and derivatives			Hordenine

CDD 8			
Fatty acids	Saturated		Tetradecanoic acid; Hexadecanoic acid; Octadecanoic acid
	Unsaturated	omega-6	9,12-Octadecadienoic acid
		omega-7	11-Octadecenoic acid
		omega-9	9-Octadecenoic acid
Terpens and terpenoids	Monoterpenes and derivatives		Citronellol

CDD 9			
Fatty acids	Saturated		Hexadecanoic acid; Octadecanoic acid
	Unsaturated	omega-6	9,12-Octadecadienoic acid
		omega-7	11-Octadecenoic acid

CDD 10			
Fatty acids	Saturated		Tridecanoic acid; Hexadecanoic acid; Octadecanoic acid
	Unsaturated	omega-6	9,12-Octadecadienoic acid
		omega-7	11-Octadecenoic acid
		omega-9	9-Octadecenoic acid
Alcohols			9-Octadecen-1-ol

CDD 11			
Fatty acids	Saturated		Dodecanoic acid; Pentadecanoic acid; Hexadecanoic acid; Octadecanoic acid
	Unsaturated	omega-6	9,12-Octadecadienoic acid
		omega-7	11-Octadecenoic acid
		omega-9	9-Octadecenoic acid
Alcohols			9-Octadecen-1-ol
Terpens and terpenoids	Monoterpenes and derivatives		Citronellol; Pinanol

CDD 12			
Fatty acids	Saturated		Hexadecanoic acid; Octadecanoic acid; Pentacosanoic acid; Triacontanoic acid
	Unsaturated	omega-6	9,12-Octadecadienoic acid
		omega-7	11-Octadecenoic acid
		omega-9	9-Octadecenoic acid
